# Middlesex Hospital

**Published:** 1832-05

**Authors:** 


### HOSPITAL REPORTS.

### MIDDLESEX HOSPITAL.

*Dropsy treated with Elaterium, &c.* By Dr. Wilson.

Case I. Lydia Ellis, aged twenty-nine, married, admitted 13th December. Ill three months with pain of chest and general swelling: had her second child 22d January last, but the swelling did not begin till the last three months, at which time a hardness was felt in the right inguinal region; pulse small and very frequent; tongue furred; bowels open; urine scanty, high coloured, acid, not albuminous; orthopnœa; fluctuation perceptible in the abdomen.—Ext. Hyoscyami, gr. v. omni nocte. Decoct. Spartii co., Inf. Digitalis, equal parts, with Spir. Æth. Nitrici, ʒi. three times a day; to which was afterwards added Potassæ Acet. ℈i.

Dec. 19th. Omit med. One grain of Elaterium dissolved in ʒiv. Spir. Æth. Nitrici; ʒi. of which to be taken every second hour, till the bowels are opened freely.

21st. Repeat Ext. Elaterium.

23d. Imperial drink.

24th. Increase the Ext. Elaterium to half-grain dose, in Spir. Æth. Nit., and to be repeated three times, if necessary.

28th. Repeat Elaterium as before.

30th. Neither diuretics nor elaterium have produced any sensible diminution of the swelling: she seems much the same as when she came in.—Fiat paracentesis abdominis.

January 2d. Three gallons of very clear water were drawn off.

4th. Extracti Elaterii, gr. ⅓, formâ pilulæ, secundis horis, ad tertiam vicem, si opus sit.

5th. Ext. Colocynth. co. gr. x. statim.

6th. Olei Crotonis Tiglii ♏i. formâ pilulæ, et enema commune statim. Repet. Ol. Crot. ♏i.

7th. Bowels freely opened this morning, for the first time since she was tapped: greatly relieved; pulse 100.—Potassæ Hydriod. ʒss. ex Aquæ ter die. Gin, ʒiij. daily. Ext. Colocynth. comp. gr. x. statim.

10th. Vomiting severe.—Omit med. Acidi Hydrocyanici ♏ij. tertiis horis, ad tertiam vicem.

11th. Ext. Colocynth. co. gr. x. statim, et rep. si opus sit.

13th. Olei Crotonis Tiglii ♏i.

14th. Repet. Ol. Crot. ♏i.

16th. Severe vomiting still continues; evacuations rare, and very scanty; has vomited several times stercoraceous matter, and relieved by it.—Olei Crotonis ♏i. formâ pilulæ, et repet. sextis horis, ad tertiam vicem, si opus sit. Enema Decocti Avenæ cum Olei Terebinthinæ, ʒvj. octavis horis repetendum. Linimentum Saponis comp. abdomini infricatum.

17th. Vomiting absent the last thirty-six hours; has had two scanty solid motions this morning.—Repet. Ol. Crot. ♏j. ter die formâ pilulæ.

18th. Has had two free evacuations this morning, in appearance natural; no sickness; much relieved.—Repet. Ol. Crot. ♏j. cras mane, et Enema bis die.

19th. A slight motion last night and this morning; appetite improved; no pain, excepting in making water, which is very scanty.

20th. Had a copious solid motion last night; vomiting returned this morning.—Repet. Pil. et Enema bis die.

21st. Vomiting continued all yesterday, ceased today; bowels not opened. At night felt much fatigued; took her gin; soon after vomited, and shortly afterwards expired.

No post-mortem examination was allowed. From the dropsy being almost entirely confined to the abdomen, and a swelling first perceived in the right inguinal region, and as, after paracentesis, the right region was more full than the left, and neither diuretics nor purgatives had produced any sensible effects, it was supposed that the dropsy was ovarian.

After being tapped, the hydriodate of potass was given as a diuretic; but, from the almost constant sickness before and afterwards, and the great difficulty now of stimulating the bowels to action, it was soon abandoned; and to effect the latter object, powerful means were had recourse to. The matter vomited at different times, about the 16th January, was certainly stercoraceous, as far as the evidence of sight and smell could be trusted; and sometimes the quantity vomited was very considerable.

Case II. A female, single, aged thirty-five, admitted 3d of February: confined eight months ago, and has been ill nearly ever since; weaned child three months since. Two months ago, head and face began to swell; a month after, swelling of abdomen commenced; and a week after that, legs began to swell. At present, swellings of face and abdomen much subsided, but legs and thighs very much swollen, and skin in some parts red; pulse full.—To be bled to ℥xvj. One third of a grain of Ext. Elaterium, made into a pill, to be taken next morning, and to be repeated every second hour for three times, should the bowels not act.

Feb. 4th. Purged and vomited by the first dose, which was not repeated.—To have one third of a grain of Elaterium only once, every second day.

8th. Dejections have been copious and liquid, but complains of much sickness and distress, caused by medicine: says her bowels are easily acted on, and begs for a less dose of medicine.—To have a quarter of a grain of Ext. Elaterium every second day.

13th. Has continued to have copious fluid motions; œdema of legs much less; catamenia have appeared, and very profuse. —Omit med. To have Potassæ Hydriodatis, ℈i. ex Aquâ, ter quotidie.

For the two first days after taking the above, the quantity of urine was not increased, but on the third day the quantity very much increased, and continued so till the swellings entirely disappeared; and the same treatment was pursued till the 27th, when she left the hospital.

Case III. John M'Cay, age twenty-one; ill six months. His feet had always perspired greatly, till one day he put them in cold water; since which, perspiration has ceased, and the feet, ankles, and face, became swollen, and have continued so ever since. Now the legs, thighs, penis, and scrotum, are greatly swollen; abdomen slightly, face more so; bowels costive; urine scanty; pulse ninety, and full.—Venæsectio ad ℥xx. Ext. Elaterii, gr. $\frac{1}{4}$; Spir. Æth. Nitrici, ʒi., ex Aquâ, to be repeated every second hour for three times, if not purged; and the same quantity to be taken every other day.

28th. Frequently vomits after medicine, but still a sufficient quantity remains to purge him. Legs and abdomen less.—To take same quantity of Elaterium made into pills.

January 2d. The doses to be increased to one third of a grain.

7th. Swellings of face, abdomen, and inferior extremities, much diminished.

18th. Has been purged every other day by elaterium: swellings almost gone.

He continued the same medicine till the 27th, after which no more elaterium was given, as he had then a sharp attack of bronchitis, for which he was twice blistered, and once took ten grains of calomel: for some days he took Mist. Amygdalæ, ℥iss.; Potas. Nitratis, gr. x.; Vini Antim. Tartarisati, ♏xx. ter die.

Feb. 6th. Much pain in the loins, with hæmaturia.—To be cupped on loins to ten ounces.

8th. Pains of loins almost gone; no hæmaturia.

Afterwards his bowels were merely regulated; and half an ounce of castor oil was generally sufficient for that purpose.

20th. Went out well.

Although the disease, in the two last cases, had been of some standing previous to the admission of the patients, they were considered as examples of inflammatory dropsy, and in both these instances bleeding was had recourse to. It may be observed, that one quarter of a grain of elaterium produced a more purgative effect on the woman than one grain did on the man. In the use of this medicine great caution is required, and the doses should at first be small; for, in addition to the difference of susceptibility to its action in different constitutions, the preparation itself varies very greatly in strength. The elaterium used in the above instance was procured from Garden's, in Oxford street.

